# Influence of COVID-19 Restrictions on Patients’ Self-reported Oral Health Care Needs

**DOI:** 10.3290/j.ohpd.b1693919

**Published:** 2021-07-15

**Authors:** Caroline Sekundo, Eva Langowski, Cornelia Frese

**Affiliations:** a Dentist, Clinic for Oral, Dental and Maxillofacial Diseases, University Hospital Heidelberg, Department of Conservative Dentistry, Heidelberg, Germany. Investigation, data curation, formal analysis, wrote original draft of the manuscript.; b Dentist, Clinic for Oral, Dental and Maxillofacial Diseases, University Hospital Heidelberg, Department of Conservative Dentistry, Heidelberg, Germany. Investigation, data curation, wrote, reviewed and edited the manuscript.; c Professor, Clinic for Oral, Dental and Maxillofacial Diseases, University Hospital Heidelberg, Department of Conservative Dentistry, Heidelberg, Germany. Conceptualisation, supervision, wrote, reviewed and edited the manuscript.

**Keywords:** COVID-19, oral health, pandemics

## Abstract

**Purpose:**

To assess how current COVID-19 restrictions regarding elective dental procedures influence patients’ self-reported oral health care needs at a University Hospital in Germany.

**Materials and Methods:**

Patients with COVID-19 induced cancellation of elective treatment appointments previously scheduled for the period March 16th to April 30th 2020 were contacted by telephone and questioned about the occurrence of oral health problems, pain, self-reported treatment needs, and the use of emergency dental services. Data were analysed retrospectively.

**Results:**

Information on 370 patients aged between 1 and 91 years was included. 16.2% (n = 60) of patients reported having experienced an oral health problem for which they requested timely dental treatment. Within this group, the most frequent complaints were pain or tooth hypersensitivity (42.4%, n = 26), insufficient restorations (28.8%, n = 17) and gingival or periodontal problems (23.7%, n = 14). Associations between the type of treatment pending and the report of an oral health problem were considerable for patients awaiting treatment under full anesthesia, surgical procedures and endodontic treatment (p = 0.001; 0.003 and 0.048, respectively). Problems were reported most frequently in these treatment groups, ranging from 27.7% to 100%, compared to 12.6% among patients scheduled for routine check-ups. Overall, 8.6% (n = 32) were experiencing pain, of whom 5 patients experienced constant pain. However, only 1.9% (n = 7) of patients made use of emergency dental services.

**Conclusions:**

The results suggest that while the postponement of routine check-ups is justifiable during emergency situations, the long-term cancellation of surgical and endodontic therapies must be viewed critically.

Emerging and re-emerging pathogens are global challenges for public health.^9^ The current SARS-CoV-2 virus outbreak, causing the COVID-19 disease, was declared a pandemic on March 11, 2020 by the World Health Organization (WHO).^4^ Its fast spread as well as the continuing change in its dynamic have led to a variety of protective measures, aiming to shield the population and secure vital processes such as health care services. Although information on the possible routes of transmission of the SARS-CoV-2 virus is still changing, the virus is known to be transmitted mainly through respiratory droplets or by contact.^3^ However, transmission of the virus may also occur through airborne transmission of aerosols.^1,20,24^ Aerosols can accumulate and remain infectious in the air for a prolonged period. Since many dental procedures produce aerosol containing the patient’s saliva or blood and take place in very close proximity to the patient’s face, dental professionals are generally thought to have a high risk of exposure.^10,14,22^ Although a recent study has shown that COVID-19 prevalence was low among practicing US dentists,^6^ concluding that the use of personal protective equipment and adherence to current infection control recommendations has proven to be effective, this information was not available at the beginning of the pandemic. It is for this reason, as well as due to mandatory resource-allocation regulations such as apply to protective equipment, that dental services were mostly reduced to emergency services early in the SARS-CoV-2 virus spread in Germany, as well as in many other countries.^2^

At the Department of Conservative Dentistry, Heidelberg University, emergency dental services were continuously provided, i.e. patients requesting treatment due to pain, trauma or serious infections such as dental abscesses were admitted. Other types of treatment were considered elective, and these treatment procedures were suspended from mid-March to the end of April 2020 in order to reduce the risk of infection and transmission of the SARS-CoV-2 virus. In addition, the postponement of the academic semester at the Heidelberg School of Dental Medicine led to the cancellation of dental services performed by dental students, who also contribute substantially to the amount of oral health care services provided. All appointments scheduled for this period were cancelled, leading to a previously unknown temporary reduction of dental care. Patients were informed about emergency dental services. It must be noted, however, that the number of patients making use of emergency dental services in the department also decreased. This illustrates the fact that, at least in part, dental appointments were also actively avoided by patients themselves, either generally out of fear of contracting COVID-19 or based on the uncertainty induced by the cancellation of their appointment and the knowledge that no regular treatments were taking place. This avoidance behavior in fear of COVID-19 has been described not only for the use of dental emergency services^12^ but also for a number of medical disciplines and medical care in general.^5,15,17^

As a result of this decline, it might seem that many dental treatments can be postponed without consequence, raising the question of the relevance of dentistry as a medical discipline, and of the value of oral health care. Whereas the financial implications of the decline in patients in the dental sector have already been analyzed in a number of studies.^7,8,21,23^ the consequences for patients’ oral health care needs have not yet been addressed.

Due to this first-time situation, it is currently unclear which potentially negative effects the cancellation and postponement of a large number of dental treatments have had on patients’ self-reported oral health and well-being. The aim of this study was therefore to assess the occurrence of oral health problems and pain experienced during the period of cancelled dental appointments, and to assess the number of patients with cancelled appointments making use of emergency dental services.

## Materials and Methods

The present study is retrospective and exploratory in design, assessing data recorded for the purpose of treatment planning. It received ethical approval by the Medical Ethics Committee of Heidelberg University (S-402/2020). The data were fully anonymised.

All elective treatment appointments (i.e. planned dental treatments for patients without severe pain, trauma or serious infections such as dental abscesses) that had been scheduled between March 16th and April 30th, 2020, were cancelled due to COVID-19 restrictions. After the specified period, as restrictions were eased and elective treatments could be performed under stricter protective and hygienic measures, patients whose appointments had been cancelled were contacted anew by the dental assistant or dental student responsible for the patient’s scheduling and interviewed via phone. Difficulties regarding their self-reported oral health-care needs and their cancelled appointments were assessed in order to reschedule appointments, if necessary, and recorded in a standardized manner (Table 1). In the case of minors, a parent was questioned.

**Table 1:** Telephone protocol: items recorded

Occurrence of an oral health problem for which the patient requires professional advice / dental treatment	Yes	No
If yes, note type of dental problem:
Occurrence of pain	Yes	No
In constant pain	Yes	No
Use of emergency services and antibiotics	Yes	No
Type of dental treatment pending:		
Check-up and professional tooth cleaning	Yes	No
Conservative restorative treatment	Yes	No
Periodontal treatment	Yes	No
Prosthetic treatment	Yes	No
Endodontic treatment	Yes	No
Surgical treatment	Yes	No
Treatment under full anesthesia	Yes	No
Showing possible COVID-19 symptoms or confirmed COVID-19 disease	Yes	No
In contact with a SARS-CoV-2 positive person	Yes	No

Data analysis was carried out with SPSS, version 24.0 (IBM; Armonk, NY, USA)s.^16^ Mean ± SD of continuous variables and proportion and frequency of categories of factor variables are reported. Differences between sociodemographic and dental variables were assessed using the Mann-Whitney U-test in the case of two categories and the Kruskal-Wallis test in the case of > 2 categories. Associations between nominal variables were analysed using Pearson’s chi-squared test or Fisher’s exact test, as appropriate. p-values are descriptive and regarded statistically significant if ≤ 0.05.

## Results

### General Data

A total of 370 patients whose appointments had been cancelled were included in this study, of which 50% (185) were women. The mean age was 46.6 (SD = 22.7) (age range: 1-91 years). No patient reported symptoms consistent with a COVID-19 infection or a positive test for SARS-CoV-2 virus. Only one patient reported to have had a confirmed contact with a SARS-CoV-2-positive person. Most patients had previously been scheduled or received treatment planning for a dental check-up and professional tooth cleaning (38.6%, n = 143) or conservative restorative treatment (32.2%, n = 119). 20.8% (77) should have received periodontal treatment, 9.7% (36) prosthetic restorations, 9.7% (36) endodontic treatment, 1.4% (5) surgical treatment and 1.1% (4) should have received conservative treatment under full anesthesia. Results may exceed 100% as multiple options were possible.

### Self-reported Oral Health Care Needs, Occurrence of Pain and Use of Emergency Dental Services

**Fig 1:**
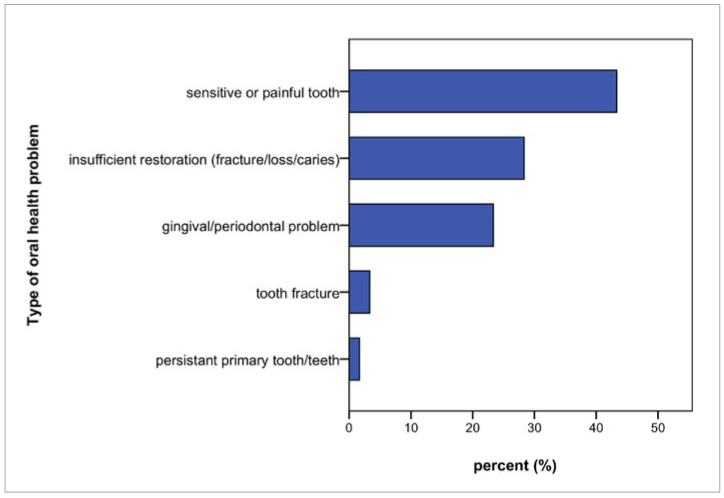
Type of oral health problem reported (n = 60).

In total, 16.2% (60) experienced an oral health problem for which they requested near-term dental treatment. Women were more frequently reported a problem than did men (p = 0.049, 20.0% vs 12.4%). There was no statistically significant difference in age regarding the occurrence of an oral health problem (p = 0.46). The most frequent complaint was pain or tooth sensitivity (43.3%, n = 26), followed by insufficient restorations due to suspected fracture, loss of the restoration or suspected cavitation/caries (28.3%, n = 17), and gingival or periodontal problems (23.3%, n = 14). There was no statistically significant difference in age or gender regarding the type of oral health problem. Figure 1 shows the relative frequency and distribution of oral health problems reported. Overall, 8.6% (32) were experiencing pain at the time of contact, of whom 15.6% (5) described the pain as constantly present. In total, 1.9% (n = 7) patients made use of emergency dental services, of whom all had reported an oral health problem, but only 5 of whom had reported pain (5/7, 71.4%) and 4 had reported this pain to be constant (4/7, 57.1%). Four patients (1.1%) received antibiotic treatment in the wake of emergency dental services.

Relative to their frequency, problems reported were most common among those originally scheduled for treatment under general anesthesia (100%, n = 4/4), surgical procedures (80%, n = 4/5), and endodontic treatment (27.7%, n = 10/36). Associations between the type of treatment pending and the occurrence of a problem were considerable in these cases (p = 0.001, p = 0.003 and p = 0.048, respectively). Amongst those scheduled for a routine check-up and prophylaxis session with no apparent treatment pending, the percentage of patients requesting dental advice or treatment reached 12.6% (18/143).

**Fig 2:**
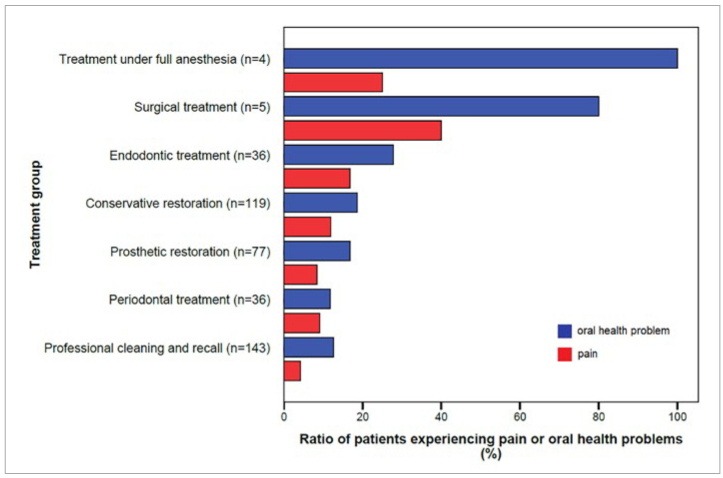
Ratio of patients experiencing pain or oral health problems among different treatment groups.

Pain was also more frequently experienced by patients scheduled for some form of treatment (11.6%) than amongst those awaiting their recall session (4.2%) (p = 0.014). Figure 2 shows the relative percentage of patients reporting an oral health problem and pain among different treatment groups.

## Discussion

This retrospective investigation analysed patient-reported data on oral health problems during the COVID-19 restrictions regarding elective dental procedures. We observed the occurrence of oral health issues in 16.2% of cases. The most frequent complaint was pain or tooth sensitivity, particularly among those awaiting treatment under full anesthesia, surgical or endodontic treatment. However, constant pain was infrequent (n = 5) and might explain why few patients made use of emergency dental services (n = 7). Of the few who did report constant pain, four out of five patients sought out emergency services.

On the one hand, this could imply that non-permanent pain is less troublesome than one might imagine and that emergency services during non-pandemic times are often misused. On the other hand, it is also possible that patients were hesitant about actively seeking dental health care services due to the transmission risks involved or insecurities caused by the cancellation of their appointment, despite requesting aid when contacted by telephone. The reasons for this behaviour were not assessed in this study, and although similar behavioural patterns (and similar considerations regarding the possible reasons) have also been reported in studies focusing on the use of emergency dental services,^13,15,18^ this question has not yet been answered and requires further research.

Several further limitations must be noted. First, our study is limited due to its retrospective and monocentric nature. Since the assessed data was not primarily collected for study purposes, the applied questions had not previously been validated. It is therefore possible that patient responses were incorrect, e.g. that patients misremembered the type of treatment pending. In addition, the use of a Visual Analogue Scale (VAS) could have improved our assessment of the reported pain. Moreover, the sole use of patient-reported data without access to additional clinical information for this study purpose prevented a more detailed analysis of the prevalence of oral health problems, e.g. between the severity of the patient’s disease and the occurrence of a problem or pain. As the study was carried out in a department of conservative dentistry (focusing on restorative, endodontic and periodontal treatments), prosthetic and surgical procedures were understandably less frequent and only small sample sizes were reached in these treatment categories.

Nevertheless, the aim of our study was primarily to assess COVID-19 restrictions from the patient’s point of view. Patient-reported data has become a fundamental element of clinical practice and research, in order to fit health care services to the needs felt by those who make use of them. In contrast to previous COVID-19 studies in the field of dentistry,^11,13,15,18,25,26^ it did not focus on the care provided by emergency services, but is the first to consider difficulties felt due to the suspension of routine dental care. Interestingly, more than one out of ten patients scheduled for routine check-ups reported an oral health problem that required professional advice or care. This underlines the relevance of regular dental appointments in the medical system and highlights the fact that oral diseases are amongst the most common diseases worldwide.^19^ The frequent report of problems amongst those awaiting endodontic treatment is in line with previous reports on the importance of endodontic problems among those consulting emergency dental services during the COVID-19 pandemic.^11,18,26^

As pointed out above, many questions remain unanswered. Further studies on the influence of the COVID-19 pandemic on dental services and oral health are necessary, including the analysis of a greater diversity of patient groups, long-term impacts and the reasons for the use or avoidance of dental services.

Although it is clear that most efforts are currently focused on the direct causes of COVID-19 and possible therapeutic options, effects on oral health resulting from cancelled appointments and reservations on the part of the patients should not be overlooked.^12^

## Conclusion

This study underlines the relevance of dental care by revealing that even patients initially only scheduled for routine dental check-ups frequently ask for dental advice. In particular, we show that a considerable number of patients awaiting treatment under full anesthesia, surgical or endodontic treatment experienced pain or other oral health problems. The results of this study therefore suggest that while the postponement of routine check-ups is justifiable during emergency situations, such as the COVID-19-pandemic, the long-term cancellation of surgical and endodontic therapies must be viewed with caution.
